# Supplementary material for: Comparison of Different Isolation Methods for Plasma-Derived Extracellular Vesicles in Patients with Hyperlipidemia
Source: Life (Basel). 2022 Nov 21;12(11):1942. doi: 10.3390/life12111942 (PMC9692895; doi:10.3390/life12111942)
Supplement: Supplementary file 1 [file life-12-01942-s001.zip › life-2010325-supplementary.pdf]

Supplementary materials

Table S1. Parameters of AF4 for the separation of plasma-derived exosomes

**Start time (min)**	**End time (min)**	**Duration (min)**	**Mode**	**Vx start (mL/min)**	**Vx end (mL/min)**
0.00	1.00	1.00	Elution	0	0
1.00	2.00	1.00	Focus	0	0
2.00	7.00	5.00	Focus+Inject	0	0
7.00	9.00	2.00	Focus	0	0
9.00	14.00	5.00	Elution	3	3
14.00	34.00	20.00	Elution	3	0.1
34.00	44.00	10.00	Elution	0.1	0

Table S2. Intensity distribution of exosomes extracted by the three methods.

UC

d (nm)	f(%)	f(cum.%)	d (nm)	f(%)	f(cum.%)	d(nm)	f(%)	f(cum.%)	d(nm)	f(%)	f(cum.%)
1.0	0.0	0.0	7.1	0.0	0.0	51.0	1.9	22.3	363.7	2.0	83.9
1.1	0.0	0.0	7.7	0.0	0.0	55.1	2.0	24.3	393.5	1.9	85.8
1.2	0.0	0.0	8.4	0.0	0.0	59.6	2.1	26.4	425.7	1.8	87.6
1.3	0.0	0.0	9.0	0.3	0.3	64.5	2.2	28.6	460.5	1.6	89.2
1.4	0.0	0.0	9.8	0.4	0.7	69.8	2.3	30.9	498.1	1.5	90.7

1.5	0.0	0.0	10.6	0.4	1.1	75.5	2.4	33.3	538.9	1.4	92.1
1.6	0.0	0.0	11.4	0.4	1.5	81.7	2.5	35.8	582.9	1.2	93.4
1.7	0.0	0.0	12.4	0.5	2.0	88.3	2.5	38.3	630.6	1.2	94.5
1.9	0.0	0.0	13.4	0.5	2.5	95.6	2.6	40.9	682.2	1.0	95.6
2.0	0.0	0.0	14.5	0.6	3.0	103.4	2.7	43.6	738.0	0.9	96.5
2.2	0.0	0.0	15.7	0.6	3.7	111.8	2.7	46.3	798.4	0.8	97.3
2.4	0.0	0.0	16.9	0.7	4.3	121.0	2.7	49.0	863.7	0.7	98.0
2.6	0.0	0.0	18.3	0.7	5.1	130.9	2.7	51.8	934.3	0.6	98.7
2.8	0.0	0.0	19.8	0.8	5.8	141.6	2.8	54.5	1010.7	0.5	99.2
3.0	0.0	0.0	21.5	0.9	6.7	153.2	2.7	57.3	1093.4	0.4	99.6
3.3	0.0	0.0	23.2	0.9	7.7	165.7	2.7	60.0	1182.8	0.4	100.0
3.5	0.0	0.0	25.1	1.0	8.7	179.3	2.7	62.7	1279.6	0.0	100.0
3.8	0.0	0.0	27.2	1.1	9.8	193.9	2.7	65.3	1384.2	0.0	100.0
4.1	0.0	0.0	29.4	1.2	11.0	209.8	2.6	67.9	1497.4	0.0	100.0
4.5	0.0	0.0	31.8	1.3	12.3	226.9	2.5	70.5	1619.9	0.0	100.0
4.8	0.0	0.0	34.4	1.4	13.7	245.5	2.5	73.0	1752.4	0.0	100.0
5.2	0.0	0.0	37.2	1.5	15.2	265.6	2.4	75.3	1895.7	0.0	100.0
5.6	0.0	0.0	40.2	1.6	16.8	287.3	2.3	77.6	2050.8	0.0	100.0
6.1	0.0	0.0	43.5	1.7	18.5	310.8	2.2	79.9	2218.5	0.0	100.0
6.6	0.0	0.0	47.1	1.8	20.3	336.2	2.1	81.9	2400.0	0.0	100.0
D(10%):	33.20	(nm)	D(50%):	60.10	(nm)	D(90%):	111.00	(nm)			

PEG

d (nm)	f(%)	f(cum.%)	d (nm)	f(%)	f(cum.%)	d(nm)	f(%)	f(cum.%)	d(nm)	f(%)	f(cum.%)
1.0	0.0	0.0	5.4	0.0	0.0	29.3	2.9	22.7	158.2	1.3	95.1
1.1	0.0	0.0	5.8	0.0	0.0	31.3	3.0	25.7	169.3	1.1	96.3
1.1	0.0	0.0	6.2	0.0	0.0	33.5	3.2	28.9	181.1	1.0	97.2
1.2	0.0	0.0	6.6	0.0	0.0	35.8	3.3	32.1	193.8	0.8	98.1
1.3	0.0	0.0	7.1	0.0	0.0	38.3	3.4	35.5	207.3	0.7	98.7
1.4	0.0	0.0	7.6	0.0	0.0	41.0	3.5	39.0	221.8	0.5	99.3
1.5	0.0	0.0	8.1	0.0	0.0	43.9	3.5	42.6	237.3	0.4	99.7
1.6	0.0	0.0	8.7	0.0	0.0	46.9	3.6	46.2	253.9	0.3	100.0
1.7	0.0	0.0	9.3	0.0	0.0	50.2	3.6	49.8	271.6	0.0	100.0
1.8	0.0	0.0	9.9	0.0	0.0	53.7	3.6	53.4	290.6	0.0	100.0
2.0	0.0	0.0	10.6	0.0	0.0	57.5	3.6	56.9	310.9	0.0	100.0
2.1	0.0	0.0	11.4	0.3	0.3	61.5	3.5	60.4	332.6	0.0	100.0
2.2	0.0	0.0	12.2	0.4	0.7	65.8	3.4	63.9	355.8	0.0	100.0
2.4	0.0	0.0	13.0	0.6	1.3	70.4	3.3	67.2	380.6	0.0	100.0
2.6	0.0	0.0	13.9	0.7	2.0	75.3	3.2	70.5	407.2	0.0	100.0
2.8	0.0	0.0	14.9	0.9	2.9	80.6	3.1	73.6	435.7	0.0	100.0
2.9	0.0	0.0	15.9	1.1	4.0	86.2	3.0	76.5	466.1	0.0	100.0
3.2	0.0	0.0	17.0	1.3	5.2	92.2	2.8	79.3	498.7	0.0	100.0
3.4	0.0	0.0	18.2	1.5	6.7	98.6	2.6	82.0	533.5	0.0	100.0
3.6	0.0	0.0	19.5	1.7	8.4	105.5	2.4	84.4	570.8	0.0	100.0
3.9	0.0	0.0	20.9	1.9	10.3	112.9	2.3	86.7	610.7	0.0	100.0

4.1	0.0	0.0	22.3	2.1	12.4	120.8	2.1	88.7	653.3	0.0	100.0
4.4	0.0	0.0	23.9	2.3	14.6	129.2	1.9	90.6	698.9	0.0	100.0
4.7	0.0	0.0	25.6	2.5	17.1	138.2	1.7	92.3	747.8	0.0	100.0
5.1	0.0	0.0	27.3	2.7	19.8	147.9	1.5	93.8	800.0	0.0	100.0
D(10%):	20.70	(nm)	D(50%):	50.40	(nm)	D(90%):	126.30	(nm)			

SEC

d (nm)	f(%)	f(cum.%)	d (nm)	f(%)	f(cum.%)	d(nm)	f(%)	f(cum.%)	d(nm)	f(%)	f(cum.%)
1.0	0.0	0.0	5.6	0.0	0.0	31.0	2.2	7.3	173.0	0.6	99.6
1.1	0.0	0.0	6.0	0.0	0.0	33.3	2.7	10.0	185.3	0.4	100.0
1.1	0.0	0.0	6.4	0.0	0.0	35.6	3.2	13.3	198.5	0.0	100.0
1.2	0.0	0.0	6.8	0.0	0.0	38.2	3.7	17.0	212.6	0.0	100.0
1.3	0.0	0.0	7.3	0.0	0.0	40.9	4.2	21.1	227.7	0.0	100.0
1.4	0.0	0.0	7.9	0.0	0.0	43.8	4.6	25.7	243.9	0.0	100.0
1.5	0.0	0.0	8.4	0.0	0.0	46.9	4.9	30.6	261.3	0.0	100.0
1.6	0.0	0.0	9.0	0.0	0.0	50.2	5.2	35.7	279.9	0.0	100.0
1.7	0.0	0.0	9.7	0.0	0.0	53.8	5.4	41.1	299.8	0.0	100.0
1.9	0.0	0.0	10.3	0.0	0.0	57.6	5.5	46.6	321.1	0.0	100.0
2.0	0.0	0.0	11.1	0.0	0.0	61.7	5.5	52.1	343.9	0.0	100.0
2.1	0.0	0.0	11.9	0.0	0.0	66.1	5.5	57.6	368.4	0.0	100.0
2.3	0.0	0.0	12.7	0.0	0.0	70.8	5.3	62.9	394.6	0.0	100.0
2.4	0.0	0.0	13.6	0.0	0.0	75.9	5.1	68.0	422.7	0.0	100.0

2.6	0.0	0.0	14.6	0.0	0.0	81.2	4.8	72.9	452.7	0.0	100.0
2.8	0.0	0.0	15.6	0.0	0.0	87.0	4.5	77.4	484.9	0.0	100.0
3.0	0.0	0.0	16.7	0.0	0.0	93.2	4.1	81.5	519.4	0.0	100.0
3.2	0.0	0.0	17.9	0.0	0.0	99.8	3.7	85.2	556.4	0.0	100.0
3.4	0.0	0.0	19.2	0.0	0.0	106.9	3.3	88.5	595.9	0.0	100.0
3.7	0.0	0.0	20.6	0.0	0.0	114.6	2.8	91.3	638.3	0.0	100.0
4.0	0.0	0.0	22.0	0.4	0.4	122.7	2.4	93.6	683.7	0.0	100.0
4.2	0.0	0.0	23.6	0.6	1.0	131.4	1.9	95.6	732.4	0.0	100.0
4.5	0.0	0.0	25.3	1.0	2.0	140.8	1.5	97.1	784.4	0.0	100.0
4.9	0.0	0.0	27.1	1.3	3.3	150.8	1.2	98.2	840.2	0.0	100.0
5.2	0.0	0.0	29.0	1.8	5.1	161.5	0.8	99.1	900.0	0.0	100.0
D(10%):	33.20	(nm)	D(50%):	60.10	(nm)	D(90%):	111.00	(nm)			
